# Supplementary material for: Patients’ Clinical and Psychological Status in Different COVID-19 Waves in Italy: A Quanti-Qualitative Study
Source: Healthcare (Basel). 2023 Sep 6;11(18):2477. doi: 10.3390/healthcare11182477 (PMC10531315; doi:10.3390/healthcare11182477)
Supplement: Supplementary file 1 [file healthcare-11-02477-s001.zip › healthcare-2273624-supplementary.pdf]

healthcare

MDPI

Psychological evaluation - Quantitative part

1) National Stressful Events Survey PTSD Short Scale (NSESSS) [1,2]
2) Patient Health Questionnaire-9 (PHQ-9) [3,4]
3) Generalized Anxiety Disorder-7 (GAD-7) [5,4]

Psychological evaluation - Qualitative part

4) In which way has COVID-19 affected your work activity? (if any) ……………………………...

5) What were the RESOURCES[1] that helped you to cope with the pandemic period? ……………...

6) What were the main EMOTIONS that you experienced in the following periods.

6.1) Outbreak of the pandemic in China:

a)____________________ b)____________________ c)____________________

6.2) Outbreak of the pandemic in Italy:

a)____________________ b)____________________ c)____________________

6.3) During your own COVID-19 illness:

a)____________________ b)____________________ c)____________________

6.4) In the present time (follow-up):

a)____________________ b)____________________ c)____________________

[1] This refers to the personal resources/characteristics or contexts (work, family, social, health) that you have used or are still using to cope as best you can during the pandemic.

References

1. Kilpatrick, D.G.; Resnick, H.S.; Friedman, M.J. *Severity of Posttraumatic Stress Symptoms—Adult (National Stressful Events Survey PTSD Short Scale [NSESSS])*; American Psychiatric Association: Washington, DC, USA, 2013

2. LeBeau, R.; Mischel, E.; Resnick, H.; Kilpatrick, D.; Friedman, M.; Craske, M. Dimensional Assessment of Posttraumatic Stress Disorder in DSM-5. *Psychiatry Res.* **2014**, *218*, 143–147. https://doi.org/10.1016/j.psychres.2014.03.032.

3. Spitzer, R.L.; Kroenke, K.; Williams, J.B. Validation and Utility of a Self-Report Version of PRIME-MD: The PHQ Primary Care Study. Primary Care Evaluation of Mental Disorders. Patient Health Questionnaire. *JAMA* **1999**, *282*, 1737–1744. https://doi.org/10.1001/jama.282.18.1737.

4. Kroenke, K.; Spitzer, R.L.; Williams, J.B.W.; Löwe, B. The Patient Health Questionnaire Somatic, Anxiety, and Depressive Symptom Scales: A Systematic Review. *Gen. Hosp. Psychiatry* **2010**, *32*, 345–359. https://doi.org/10.1016/j.genhosppsych.2010.03.006.

5. Spitzer, R.L.; Kroenke, K.; Williams, J.B.W.; Löwe, B. A Brief Measure for Assessing Generalized Anxiety Disorder: The GAD-7. *Arch. Intern. Med.* **2006**, *166*, 1092–1097. https://doi.org/10.1001/archinte.166.10.1092.
